# Severe Gastroduodenitis Associated With Ulcerative Colitis After Total Colectomy Successfully Treated With Endoscopic Hemostasis and Oral Tacrolimus

**DOI:** 10.1002/deo2.70217

**Published:** 2025-10-06

**Authors:** Koji Fujimoto, Shuhei Hosomi, Yumie Kobayashi, Rieko Nakata, Yu Nishida, Fumio Tanaka, Yasuhiro Fujiwara

**Affiliations:** ^1^ Department of Gastroenterology Graduate School of Medicine Osaka Metropolitan University Osaka Japan

**Keywords:** colectomy, duodenal ulcer, pouchitis, tacrolimus, ulcerative colitis

## Abstract

Herein, we report a rare case of gastroduodenitis associated with ulcerative colitis (UC). A 42‐year‐old man was diagnosed with UC 1 year prior to admission to our hospital. The patient underwent a 3‐stage total colectomy and ileal pouch‐anal anastomosis for severe UC. Two months after the second surgery, the patient was admitted to our hospital with nausea, appetite loss, abdominal pain, and frequent bloody diarrhea. Blood analysis showed an increase in white blood cell count and C‐reactive protein levels. Esophagogastroduodenoscopy (EGD) revealed diffuse UC‐like inflammation from the stomach to the duodenum and ulcers in the descending and horizontal regions of the duodenum. Pouchoscopy revealed ulcers and friable mucosa within the pouch. The patient was diagnosed with gastroduodenitis associated with UC (GDUC) and diversion pouchitis based on endoscopic and pathological findings. Inflammation in the GDUC was resistant to oral crushed mesalazine and prednisolone (60 mg/day) infusion, resulting in arterial bleeding from the duodenal ulcer and bloody stool in the stoma. Endoscopic hemostasis was performed for the duodenal ulcer. Oral tacrolimus was initiated because the inflammation was steroid‐resistant. Approximately 2 weeks after the initiation of tacrolimus, abdominal symptoms, including bloody diarrhea, disappeared, and EGD showed improvement in the GDUC.

## 1 Introduction

Ulcerative colitis (UC)‐associated intestinal complications include gastroduodenitis associated with UC (GDUC) and pouchitis. UC is characterized by limitation of the inflammation to the colon; in GDUC, an inflammation similar to that of UC occurs in the stomach and duodenum. The incidence rate of GDUC is 4.7%–7.6% in Japan [1, 2]. In many cases, GDUC improves with UC treatment. However, GDUC may worsen when total colectomy is performed and treatment for UC is discontinued. In addition, pouchitis is a common complication of total colectomy, and patients with GDUC have a high incidence of pouchitis [2].

Treatment for GDUC has not been established, although crushed mesalazine tablets and corticosteroids have been reported to be effective [3]. Besides, there are some case reports of severe GDUC treated with tumor necrosis factor‐α (TNF‐α) inhibitors, ustekinumab, upadacitinib, and intravenous tacrolimus [4, 5, 6, 7]. Herein, we report a rare case of severe GDUC after colectomy with arterial bleeding from a duodenal ulcer that was successfully treated with endoscopic hemostasis and oral tacrolimus.

## 2 Case Report

A 42‐year‐old man was diagnosed with proctitis‐type UC approximately 1 year prior to admission to our hospital and had a medical history of bullous pemphigoid. Although the patient was treated with oral mesalazine and budesonide enema, UC progressed to severe pancolitis. The inflammation was resistant to prednisolone, upadacitinib, and mirikizumab. Oral tacrolimus was partially effective, but it did not induce remission. Therefore, the patient was scheduled for 3‐stage surgery and underwent a total colectomy and ileostomy 8 months prior to admission. The patient underwent rectal resection and ileal pouch‐anal anastomosis 2 months prior to admission. The patient had an ileostomy in place.

The patient was admitted to our hospital with symptoms of nausea, appetite loss, abdominal pain, and bloody diarrhea 2 weeks prior to admission. On admission, the patient had abdominal tenderness and bloody diarrhea occurring approximately 30 times per day. The stool in the stoma was not bloody.

**FIGURE 1:**
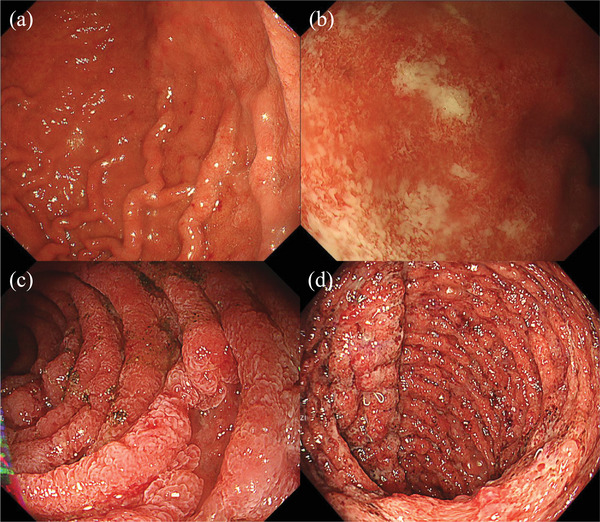
At admission, endoscopic findings of the stomach (a), the duodenal bulb (b), the duodenum with horizontal lesion (c), and the pouch (d).

**FIGURE 2:**
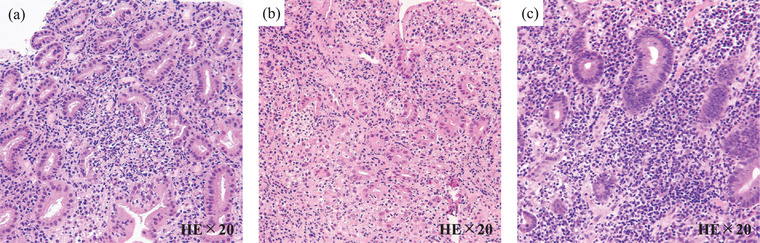
Pathological findings showing focally enhanced gastritis (a), crypt abscess and cryptitis of the duodenum (b), and crypt abscess and cryptitis of the pouch (c).

Blood analysis showed anemia (hemoglobin 12.7 g/dL), hypoalbuminemia (Alb, 2.8 g/dL), and elevated white blood cell count (WBC, 14,500 /µL) and C‐reactive protein (CRP; 5.47 mg/dL) levels. Esophagogastroduodenoscopy (EGD) showed reddened mucosa and multiple aphthae from the body to the antrum of the stomach and granular and friable mucosa from the bulb to the horizontal region of the duodenum. Ulcers were confirmed in the descending and horizontal regions of the duodenum (Figure 1a–c). Pouchoscopy revealed ulcers and friable mucosa in the pouch (Figure 1d). Endoscopy of the entire small intestine, such as capsule endoscopy and double‐balloon endoscopy, was not performed because ileoscopy through the stoma revealed that the ileal mucosa on the oral side of the stoma was normal. Serum antibody test results for *Helicobacter pylori* were negative. Stool cultures were negative for bacterial infection and *Clostridioides difficile* antigens and toxins. Cytomegalovirus (CMV) infection was not confirmed via mucosal biopsy, and CMV antigenemia (C7HRP) was negative. Pathological findings revealed focally enhanced gastritis and crypt abscess, and cryptitis in the duodenum (Figure 2a–b). Mucosal biopsy of the pouch revealed crypt abscesses and cryptitis (Figure 2c). Based on endoscopic and pathological findings, the patient was diagnosed with GDUC and diversion pouchitis.

**FIGURE 3:**
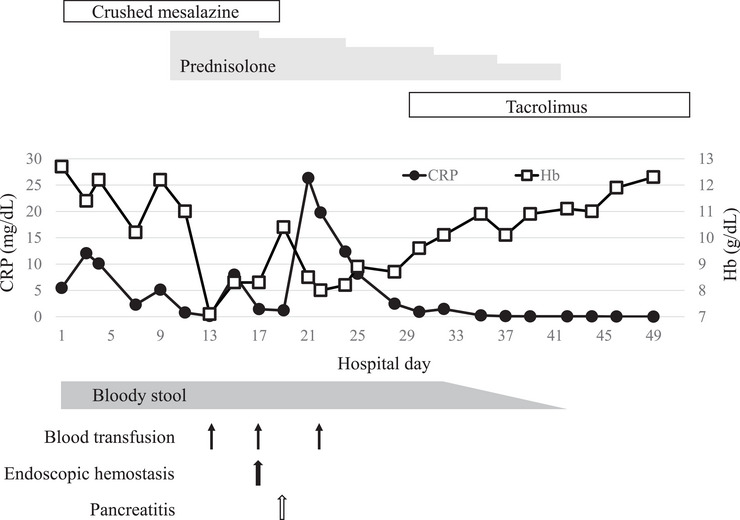
Clinical course of the patient. CRP; C‐reactive protein, Hb; hemoglobin.

**FIGURE 4:**
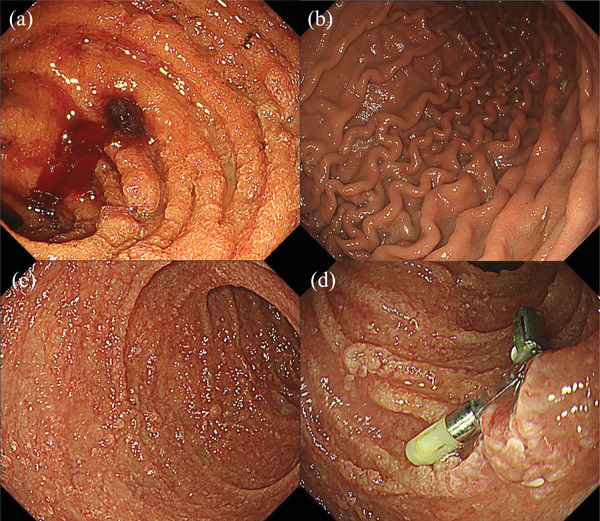
Endoscopic finding of arterial bleeding from the duodenal ulcer of the horizontal region on day 17 of admission (a). Endoscopic findings of the stomach (b), duodenal bulb (c), and duodenum with horizontal lesion (d) on day 45 of admission.

The patient fasted and was treated with orally crushed mesalazine (2000 mg/day) and budesonide enema. However, these treatments were ineffective and did not alleviate the patient's symptoms. Therefore, intravenous prednisolone (60 mg/day) was initiated on day 9 after admission (Figure 3). On day 17 of admission, EGD was performed because of progressive anemia requiring multiple blood transfusions due to increased bloody stools in the stoma. EGD revealed arterial bleeding from the duodenal ulcer in the horizontal region (Figure 4a). Endoscopic hemostasis was achieved using clips. On day 19 of admission, oral mesalazine was discontinued because of drug‐induced pancreatitis caused by mesalazine. On day 29 of admission, pouchoscopy revealed that diversion pouchitis did not improve. Owing to the worsening of the duodenal ulcer and lack of improvement in diversion pouchitis, oral tacrolimus (6 mg/day) was administered on day 29 of admission. The tacrolimus dose was adjusted based on the trough level. The symptoms gradually improved after the initiation of oral tacrolimus. CRP levels remained negative after day 37 of admission. On day 45 of admission, EGD revealed scarring from the duodenal ulcer, and the GDUC improved (Figure 4b–d). Relapses of GDUC and diversion pouchitis were not confirmed after meal resumption, and the patient was discharged on day 51 of admission. The patient switched from tacrolimus to ustekinumab after discharge and remained in clinical remission.

## 3 Discussion

UC generally involves inflammation limited to the large intestine; however, inflammation can occur in areas other than the large intestine, such as in cases of GDUC, pouchitis, and backwash ileitis. In our case, the simultaneous occurrence of GDUC and diversion pouchitis following total colectomy with ileal pouch‐anal anastomosis highlights the systemic and immune‐mediated nature of UC. Although the etiologies of GUDC and pouchitis remain unknown, Honma et al. reported that nodular duodenitis involves CD8^+^ cell infiltration in patients with UC [8], suggesting that immunoregulatory abnormalities extend beyond the colon, possibly due to abnormal T‐cell responses and loss of regulatory control of the mucosal immune system. Similar to the mechanisms of GUDC pathogenesis, impaired barrier function and inappropriate innate or adaptive immune responses with dysbiosis‐induced mucosal immune activation are considered central to the pathogenesis of pouchitis in patients with UC [9]. Previous studies reporting that the occurrence of GDUC was correlated with the occurrence of pouchitis support the involvement of immunologic mechanisms in the pathogenesis of both conditions [1, 2]. In our case, diversion pouchitis was more severe than GDUC at the time of admission. Therefore, diversion pouchitis may have caused a systemic immune response associated with UC and resulted in GDUC. Hori et al. reported that a longer postoperative period was one of the characteristics of GDUC because prednisolone is tapered after surgery [1]. Discontinuing treatment for UC after surgery may have affected the onset of GDUC and diversion pouchitis.

Although the treatment for GDUC has not been established, crushed mesalazine and corticosteroids are often administered first [3]. In steroid‐refractory cases, advanced therapies, such as TNF‐α inhibitors, ustekinumab and upadacitinib, should be administered according to the treatment of UC [4, 5, 6]. Although there have been case reports of GDUC treated with intravenous tacrolimus [7], to the best of our knowledge, this is the first case report of severe GDUC in which bleeding duodenal ulcer was successfully treated with endoscopic hemostasis and oral tacrolimus.

In the present case, induction therapy with tacrolimus or infliximab was considered according to the treatment for acute severe UC because GDUC and diversion pouchitis were severe. Tacrolimus was prescribed because tacrolimus was partially effective for UC before surgery, and TNF‐α inhibitors are associated with the risk of worsening of bullous pemphigoid [10]. If tacrolimus did not improve the inflammation within 2 weeks, another treatment such as Janus kinase inhibitors or ustekinumab was considered. Tacrolimus, a calcineurin inhibitor that suppresses interleukin‐2‐mediated T cell activation, is effective in corticosteroid‐resistant UC. In our case, tacrolimus induced the remission of GDUC and pouchitis, suggesting that T‐cell‐mediated immune pathogenesis may be common to both processes. Ustekinumab was initiated as maintenance therapy after induction therapy with tacrolimus. EGD performed approximately 1 year after discharge showed that the GDUC remained in remission.

This case highlights two crucial clinical implications. First, it underscores the importance of early recognition and endoscopic evaluation in patients with UC and upper abdominal symptoms following total colectomy. Second, it demonstrates the potential efficacy of oral tacrolimus as a rescue therapy not only in steroid‐resistant severe pouchitis but also in GDUC.

In conclusion, this is the first case report of severe GDUC after total colectomy that was successfully treated with endoscopic hemostasis and oral tacrolimus. In patients undergoing total colectomy for UC, early EGD should be considered if upper abdominal symptoms are present. Advanced therapies, including tacrolimus therapy, should be considered in cases of severe GDUC resistant to prednisolone.

## Author Contributions

**Koji Fujimoto**, **Shuhei Hosomi**, **Yumie Kobayashi**, **Rieko Nakata**, **Yu Nishida**, **Fumio Tanaka**, and **Yasuhiro Fujiwara** drafted the study. **Koji Fujimoto** performed data analysis and wrote the paper. **Koji Fujimoto**, **Shuhei Hosomi**, **Yumie Kobayashi**, **Rieko Nakata**, **Yu Nishida**, **Fumio Tanaka**, and **Yasuhiro Fujiwara** revised the manuscript. All authors read, commented on, and approved the final version of the manuscript.

## Conflicts of Interest

The authors declare no conflicts of interest.
